# Study of prevalence, distribution and clinical significance of *Blastocystis* isolated from two medical centers in Iran 

**Published:** 2017

**Authors:** Tahereh Rezaei Riabi, Ali Haghighi, Hamed Mirjalali, Sara Mohammad Ali Gol, Seyed Ahmad Karamati, Mehrdad Ghasemian, Ayat Bahadori Monfared, Elham Aghamohammadi, Homayoun Zojaji

**Affiliations:** 1 *Department of Medical Parasitology and Mycology, Faculty of Medicine, Shahid Beheshti University of Medical Sciences, Tehran, Iran.*; 2 *Foodborne and Waterborne Diseases Research Center, Research Institute for Gastroenterology and Liver Diseases, Shahid Beheshti University of Medical Sciences, Tehran, Iran.*; 3 *Behbood Research Center for Gastroenterology and Liver Diseases, Shahid Beheshti University of Medical Sciences, Tehran, Iran.*; 4 *Imam Hossein Hospital, Shahid Beheshti University of Medical Sciences, Tehran, Iran.*; 5 *Faculty of Medicine, Shahid Beheshti University of Medical Sciences, Tehran, IR Iran*; 6 *Basic and Molecular Epidemiology of Gastrointestinal Disorders Research Center, Research Institute for Gastroenterology and Liver Diseases, Shahid Beheshti University of Medical Sciences, Tehran, Iran.*; 7 *Gastroenterology and Liver Diseases Research Center, Research Institute for Gastroenterology and Liver Diseases, Shahid Beheshti University of Medical Sciences, Tehran, Iran *

**Keywords:** Iran, Blastocystis, Prevalence, Age, Clinical manifestation

## Abstract

**Aim:**

This study aimed to survey prevalence and clinical significance of *Blastocystis* among symptomatic and asymptomatic groups.

**Background:**

*Blastocystis* is a prevalent microorganism that is found in intestine of human and majority of animals. However, most studies have failed to establish correlation between the presence of the parasite and clinical manifestations.

**Methods:**

from Dec 2016 to Jun 2017, 554 stool samples were collected from symptomatic and asymptomatic subjects referred to Imam Hossein Hospital and Gastroenterology and Liver Diseases Research Institute, Tehran, Iran. All samples were concentrated using conventional formalin-ethyl acetate concentration and then were microscopically examined using Lugol’s iodine staining and light microscope. The fresh stool samples were also cultivated in DMEM medium and were examined for growth of *Blastocystis* every 48 hours with direct smear slides for 10 days.

**Results:**

*Blastocystis* was observed among 93 (16.8%) of stool samples cultivated in DMEM. The findings represented that 64/398 (16.08%) and 29/156 (18.58%) of asymptomatic and symptomatic patients were infected with *Blastocystis*, respectively. In addition, there was no significant correlation between presence of symptoms and carrying *Blastocystis* (P=0.528), although statistically significant association was observed between presence of urticaria and carrying *Blastocystis* (P<0.05). Furthermore, a statistically significant correlation between observing the parasite and different age groups was seen (P<0.05).

**Conclusion:**

*Blastocystis* is a prevalent parasitic eukaryote among symptomatic and asymptomatic populations despite the higher prevalence among symptomatic group that suggests the chance of infection with *Blastocystis* raises with age.

## Introduction

*Blastocystis *is the most common anaerobic intestinal parasite that frequently isolated from human and large variety of vertebrates. This parasitic protozoan firstly described in the early 1900s (1, 2); however, pathogenicity of *Blastocystis *has remained controversial, so far (3-5). Although cyst stage is known as the most probable transmission form of the parasite, human to human and zoonotic transmission are the main transmission routs (1). According to the available epidemiologic investigations, *Blastocysti*s is reported with a higher prevalence in low-income countries (6, 7). However, high prevalence of the parasite among those populations that have close contact to animals notifies the public health point of view of this interesting eukaryote (8). 

As it is mentioned by available literatures, pathogenicity of this parasitic eukaryote has not been illustrated, although there are reports of *Blastocystis *from patients with gastrointestinal and ex-gastrointestinal disorders (9). Although the symptoms are mostly nonspecific, diarrhea, abdominal pain, nausea, vomiting and flatulence have frequently reported by infected subjects (10-13).

Genetically, *Blastocystis *is a highly divergent eukaryote that based on remarked nucleotide diversity throughout small subunit ribosomal RNA gene, 17 separated linkages (known as subtype/ST) have characterized from human, domesticated animals, birds and even reptiles (14-17). From the identified subtypes, ST1-9 are the common subtypes reported from human subjects of which ST3 is known as the predominant subtype (2). Although several studies have tried to establish a linkage between presence of certain subtypes and clinical manifestations, only rare studies have shown a statistically significant correlation. However, some occasional reports have revealed correlation between presence of subtypes 1 and 4 with gastrointestinal disorders (11, 18, 19). 

During the last decades, several studies have described *Blastocystis *from symptomatic and asymptomatic patients using molecular approaches such as PCR RFLP (20), sequences tagged-size primers (STS) (21, 22) and sequencing of SSU rRNA gene (23-25) indicating high prevelance of *Blastocystis *in Iran. In the current study, we aimed to assess prevalence, distribution and clinical data of *Blastocystis *obtained from stool samples of symptomatic and asymptomatic subjects using cultivation and microscopical examination. 

## Methods

**Sample collection**

During December 2016 to June 2017, 554 stool samples were collected from symptomatic and asymptomatic patients who admitted to Gastroenterology and Liver Diseases Research Institute and Imam Hossein hospital. A trained interviewer filled questioners consisted of demographic data and also medical history. All samples were immediately transferred to the parasitology laboratory located at Foodborne and Waterborne Diseases Research Center, Gastroenterology and Liver Diseases Research Institute, Shahid Beheshti University of Medical Sciences, Tehran, Iran. 

**Cultivation**

All samples were divided into two portions; a portion was concentrated using routine formalin-ethyl acetate concentration and then were microscopically examined using Lugol’s iodine staining and light microscope (magnification X400). Another portion of samples was cultivated in Dulbecco's Modified Eagle Medium (DMEM) (Gibco, Thermo Fisher Scientific, MA, USA) supplemented with 10% Penicillin-Streptomycin 20% and inactivated horse serum (Gibco). Briefly, approximately 200 mg of stool samples was inoculated in DMEM medium and then the cultivated samples were incubated at 37 °C in an anaerobic condition and were examined every 48-72h for 10 days. The sample without any growth of *Blastocystis *after 10 days was considered as negative. In addition, the positive samples were sub-cultivated every 3-4 days.

**Statistical analysis**

In order to evaluate the probable correlation between presence and type of symptoms as well as age with carrying *Blastocystis,* Fisher's Exact Test incorporated in IBM SPSS Statistics for Windows, v22 (Chicago, IL, USA) was employed. A probability (P) value < 0.05 was considered statistically significant. 

## Results

From total 554 human subjects included in our study, 250 (45.1%) and 304 (54.9%) were male and female, respectively. The mean + SD of age of enrolled patients was 33.01 + 20.97 years. The results of microscopical examination, and cultivation of *Blastocystis* in DMEM medium represented that the parasite was seen among 93 (16.8%) of stool samples. Association between gender and carrying the parasite was investigated that revealed no statistically significant correlation between the presence of *Blastocystis *and gender. 

The results showed that the parasite was seen among 64/398 (16.08%) and 29/156 (18.58%) of asymptomatic and symptomatic patients, respectively. The findings showed that at least one symptom including diarrhea 116 (20.9%), constipation 59 (10.6%), nausea 105 (19%), bloating 122 (22%) and urticaria 7 (1.3%) was seen among the enrolled symptomatic subjects. The presence of *Blastocystis* was also investigated regarding type of symptoms that showed prevalence of *Blastocystis *among 19/116(16.37%), 10/59 (16.94%), 19/122 (15.57%), 15/105 (14.28%), 5/7 (71.42%) from patients with diarrhea, constipation, bloating, nausea and urticaria, respectively. Fisher’s Exact Test showed that although there was no significant correlation between presence of symptoms and carrying *Blastocystis *(*P*= 0.528), a statistically significant association was observed between presence of urticaria and carrying *Blastocystis* (*P*<0.05). All relevant data was summarized in table 1.

Correlation between age and presence of *Blastocystis *was also assessed and Fisher’s Exact Test represented a statistically significant correlation between observing the parasite and different groups of age (*P *value<0.05). Accordingly, the lowest prevalence of the parasite was seen in patients who were less than 20 years old, while the parasite was seen among an average of 20% of enrolled participants more than 20 years old (Table 1).

## Discussion

During recent years, numerous epidemiological studies have investigated prevalence of *Blastocystis* among symptomatic and asymptomatic human subjects (10, 23, 26, 27) as well as different groups of animals ranged from birds to reptiles (16, 17, 28). Accordingly, available data indicate that *Blastocystis *more likely is the most prevalent parasitic eukaryote reported from human and animals (2, 28). Along with epidemiological surveys around the world, many studies have investigated the prevalence of *Blastocystis *among symptomatic and asymptomatic patients in Iran (20, 21, 23-25, 29). In the current study, *Blastocystis *was seen among 16.3% of enrolled patients. This finding is in accordance with the prevalence rate of the parasite that previously reported from Iran. 

In a study conducted by Moosavi *et al*, *Blastocystis *was seen among 23.8% of stool samples, which were cultivated in DMEM medium, while only 15.2% of samples were obtained positive using routine microscopical examination. In this study, they claimed that stool cultivation enhances the chance of detection of *Blastocystis *from stool samples (21). In another study carried out by Badparva *et al*, only 6.5% of the samples were identified positive for *Blastocystis *(22)*. *Although in this study DNA was extracted directly from stool samples, probably PCR inhibitors of stool samples decreased the chance of detection of the parasite. This observation was confirmed by next studies performed by Jalallu (23) and Salehi (13), showing high sensitivity of culture media for detection of *Blastocystis *from stool samples. In the current study, a comparison between different methods of detection was not performed, but the finding showed that cultivation of stool samples not only enhanced the chance of detection of *Blastocystis *from stool samples, but also may decrease the effects of PCR inhibitors for further molecular studies.

The current findings showed that there was no statistically correlation between report of the parasite and gender. Although there were studies indicating statistically association between gender and carrying *Blastocystis*, our results represented no association between gender and infection with *Blastocystis. *This finding was supported by studies performed in Iran (13), Cyprus (30), Qatar (31), Philippine (32) and Turkey (10). 

**Table1:** Prevalence of *Blastocystis *among enrolled participants regarding the demographic data

Variables	No. of samples (%)	No. of *Blastocystis-*positive samples (%)	*P*-value
Gender			
Male female	250 (45.1) 304 (54.9)	37 (14.8) 56 (18.48)	0.381
Age			
<20 years 21-35 years 36-50 years 51-65 years >65 years	178 (32.12) 154(27.79) 82 (14.8) 100(18.05) 40(7.22)	7(3.93) 36(23.37) 21(25.6) 21(21) 8(20)	< 0.05
Symptoms			
Diarrhea Constipation Bloating Nausea Urticaria	116 (20.9) 59 (10.6) 122 (22) 105 (19) 7 (1.3)	19 (16.37) 10 (16.94) 19 (15.57) 15 (14.28) 5 (71.42)	1 1 0.784 0.562 <0.05
Non-symptoms	398 (71.84)	64 (16.08)	-

Statistically correlation between age and the presence of *Blastocystis *was also assessed and showed significant association. Many studies have evaluated the correlation between age and the infection. However, most of the studies indicated highest prevalence of *Blastocystis *among participants with age more than 20 years (31-33). Jalallu and colleagues stated that highest prevalence of *Blastocystis *was seen among patient more than 70 years (23). Another survey by Seyer and colleagues showed that although statistically correlation between age group and the presence of *Blastocystis *was not seen, highest prevalence of the parasite was reported from age group older than 20 years (30). These studies together with other investigations support our data that represented high prevalence of *Blastocystis *among participants aged more than 20 years that might be resulted from higher out of home activities of participants aged more than 20 years in comparison with those subjects younger than 20 years. 

Numerous studies have tried to establish significant correlation between the presence of *Blastocystis *and clinical manifestations. Although some investigations revealed positive-correlation between some disorders and the infection (34-36), most of studies have failed to establish statistically significant association between clinical manifestations and *Blastocystis*. 

Importantly, according to the results of the current investigation, *Blastocystis *was seen among 18.58% of symptomatic patients, while the parasite was obtained from stool samples of 16.08% of asymptomatic individuals. In the current study in accordance with previous surveys, statistically correlation was not seen between presence of symptoms and carrying *Blastocystis*, although statistically association was seen between urticaria and the infection. Several studies indicated that *Blastocystis *can cause extra intestinal disorders like urticaria (37-39). Notably, this is the first study, indicating *Blastocystis *from urticaria patient in Iran. However, *Blastocystis *is considered as controversial parasite that its pathogenicity is not clear.

*Blastocystis* is a prevalent parasitic eukaryote among symptomatic and asymptomatic populations with higher prevalence among symptomatic group. Furthermore, it seems that the chance of infection with *Blastocystis* raises with age.
